# The Official Scheme for the Medical Service of the Territorial Army

**Published:** 1907-11-16

**Authors:** 


### The Royal Army Medical Corps Section.

### THE OFFICIAL SCHEME FOR THE MEDICAL SERVICE OF THE TERRITORIAL ARMY.

Too much interest cannot be taken by the medical profession generally, and especially by Volunteer medical officers, in the subject of the proposed medical arrangements for the new Territorial Army. As the main outlines of the scheme have been made public by Sir Alfred Keogh, the Director-General of the Army Medical Service, it is now possible to indicate what these are.

The first point of importance—and it is one that is sure to be very freely discussed in Volunteer medical circles—is the proposal to frame the new medical organisation on the basis of a single consolidated and homogeneous corps, to which every member of the new medical service must belong. To this idea it is possible there may be opposition on the part of some of the present regimental medical officers, but we think it is a wise step and is in the interests of the medical department of the Territorial Army. It is an arrangement that has been already advocated in the pages of The Hospital, and it is in accordance with the general wish of the present Volunteer medical officers, who have expressed themselves to that effect in the representations made by them to the authorities at the War Office. It should be remembered that such a consolidated corps is not necessarily destructive of everything that exists. It is quite compatible with the retention of what experience has shown to be good in the present Volunteer medical service; and in proof of this it may be pointed out that the bearer companies and field hospitals of the Royal Army Medical Corps (Volunteers) will be retained, and will become consolidated into field ambulances for the Cavalry and Infantry. In the same way, the existing Volunteer regimental system is not to be swept away. It is the intention to perpetuate it in a modified and improved form throughout the Territorial Army. As regards the actual details of the scheme, it is very gratifying to find that the proposed new medical service is to be in every way complete in itself. Modelled on the lines of the medical department of the Regular Army, it is to be supplied with its own administrative department, with its own hospitals, and with its own sanitary staff. In addition, by the co-operation of the nursing matrons of the civil hospitals in our large cities and towns, it is hoped to establish a nursing branch in connection with it, whose members will volunteer for duty in time of war in the territorial hospitals. All these are new and excellent features, and not only do they make for efficiency, but they open up a variety of work to suit the inclinations and the special aptitude of the different members who may join the new service. They give a choice of duties, and an individual may elect to serve as regimental surgeon, or with the field ambulance, or in hospital, or in the sanitary branch, or on the Administrative Divisional Staff, according a[illegible] his tastes lead him. It is with great satisfaction [illegible] note that special attention has been paid to the [illegible]tablishment of a sanitary branch in the proposed medical department for the Territorial Army. The value of preventive medicine to an army in the field cannot be overestimated, but especially is it called for in the case of citizen soldiers. Were any considerable force of them to be suddenly mobilised, coming as they do from their own homes, many of which are likely to be infected, they are bound to bring together the seeds of disease, and in a short time a serious epidemic might arise. It is under such conditions that the advice and assistance of our everyday officers of health would be invaluable in establishing safeguards and precautions to minimise this danger to the utmost. Posted, too, as members of the sanitary corps would be to individual regiments and units, they would exercise a watchful care over the health of the men composing them both on the line of march and in camp, and thus keep them free from sickness. The value to troops in the field of a good sanitary system was well exemplified in the Russo-Japanese war, and the lesson there taught should not be lost sight of. In fact, the war efficiency of an army may be said to depend on the excellence or otherwise of its sanitary organisation.

Very properly, too, a good deal has been done to make the conditions of service in the new organisation as little irksome as possible to those joining it. The old Volunteer regulations, by their stringency and their inflexibility, made it impossible for the majority of our leading physicians and surgeons to take part in Volunteer medical work. Under the new *régime* it is hoped they may be encouraged to do so. In the first place, it is intended that there should be no demands made upon them in peace time. It is recognised that hospital physicians and surgeons, sanitary officers, and members of the nursing service do not need any annual training. The duties they will voluntarily assume in time of war in the United Kingdom are identical with those they perform every day in civil life, and consequently no demands need be made upon them. They will become what are termed members *à la suite* of the medical service. Again, for those members who have to obtain certificates of proficiency and to undergo training in various branches of military medical work, increased facilities will be given in these two directions both by the establishment of additional schools of instruction, and by placing the resources of every military hospital throughout the country at the disposal of the medical officers of the territorial force. Further, very liberal rates of pay will be given to all members, according to rank, when called out for training.

In view of what has been said, and looking to the main features of the proposed scheme, we are of opinion that it merits favourable consideration at the hands of the medical profession, to whom it has been addressed. One very noteworthy fact in connection with it is the considerate attitude adopted in placing it before the profession. There is a welcome absence of that peremptory tone that has been in the past so characteristic of official announcements. Sir Alfred Keogh deserves credit for the course he is adopting. Desirous that those who wear the shoe should say where it may pinch them, he has asked from them their views on this matter of his proposed medical organisation, and he wishes to have from them any criticisms or suggestions they may have to offer before definitely settling the details of his plan. No doubt this offer will be taken advantage of, and some changes may be subsequently made; but whether this be so or not, it has already been made abundantly clear that the new proposals have been drawn up with an honest wish to provide a good medical service, care being taken that its requirements are in harmony with the conditions of civilian life and possess sufficient elasticity to make them workable. This is a policy that should have been long ago pursued when dealing with the Volunteers, and it is to be hoped that it is the one that will be followed with the new Territorial Army. The present Director-General is to be congratulated on adopting it in the present instance, and he may rest assured that it will have good results.

